# Design of a trial-based economic evaluation on the cost-effectiveness of employability interventions among work disabled employees or employees at risk of work disability: The CASE-study

**DOI:** 10.1186/1471-2458-12-43

**Published:** 2012-01-18

**Authors:** Cindy YG Noben, Frans JN Nijhuis, Angelique E de Rijk, Silvia MAA Evers

**Affiliations:** 1 School for Public Health and Primary Care (CAPHRI), Maastricht University, Maastricht, the Netherlands; 2 Department of Health Service Research, Maastricht University, Maastricht, the Netherlands; 3 Department of Work and Social Psychology, Maastricht University, Maastricht, the Netherlands; 4 Department of Social Medicine, Maastricht University, Maastricht, the Netherlands

**Keywords:** Economic evaluation, Cost effectiveness, Employability, Return-to-Work, Work disability

## Abstract

**Background:**

In the Netherlands, absenteeism and reduced productivity due to work disability lead to high yearly costs reaching almost 5% of the gross national product. To reduce the economic burden of sick leave and reduced productivity, different employability interventions for work-disabled employees or employees at risk of work disability have been developed. Within this study, called 'CASE-study' (Cost-effectiveness Analysis of Sustainable Employability), five different employability interventions directed at work disabled employees with divergent health complaints will be analysed on their effectiveness and cost-effectiveness. This paper describes a consistent and transparent methodological design to do so.

**Methods/design:**

Per employability intervention 142 participants are needed whereof approximately 66 participants receiving the intervention will be compared with 66 participants receiving usual care. Based on the intervention-specific characteristics, a randomized control trial or a quasi-experiment with match-criteria will be conducted. Notwithstanding the study design, eligible participants will be employees aged 18 to 63, working at least 12 h per week, and at risk of work disability, or already work-disabled due to medical restrictions. The primary outcome will be the duration of sick leave. Secondary outcomes are health status and quality of life. Outcomes will be assessed at baseline and then 6, 12 and 18 months later. Economic costs will consist of healthcare costs and cost of lost production due to work disability, and will be evaluated from a societal perspective.

**Discussion:**

The CASE-study is the first to conduct economic evaluations of multiple different employability interventions based on a similar methodological framework. The cost-effectiveness results for every employability intervention will be published in 2014, but the methods, strengths and weaknesses of the study protocol are discussed in this paper. To contribute to treatment options in occupational health practice and enable the development of guidelines on how to conduct economic evaluation better suited to this field; this paper provides an important first step.

**Trial registration:**

Four trials involved in the CASE-study are registered with the Netherlands Trial Registry: Care for Work (NTR2886), Health and Motion (NTR3111), Guidance to Excel in Return to Work (NTR3151), Care for Companies/Second Care (NTR3136).

## Background

### Work disability

Many recent studies have focused on reduced productivity and work absence due to work disability in Western countries [1-5]. Work disability prevents or detracts the employee's productivity and has many economic and public health consequences, such as problems for employers, employees, their families, and society [6-8]. In Western countries its main consequences are financial, due to reduced productivity, increased job turnover and the cost of additional rehabilitation programs [9-11]. In order to estimate the costs of work disability, staff absence and presence needs to be recorded. Absenteeism refers to the total days lost from work. Presenteeism refers to attending work whilst still stick or disabled, causing reduced productivity while at work [12]. Absenteeism and presenteeism can be used as integrated measures of physical, psychological and social functioning in studies of working populations [12,13]. In the Netherlands, the Sickness Absence Reduction Act was introduced in 1994 in an attempt to reduce absenteeism. Although there has been a major reduction in absenteeism, nearly 3 million Dutch workers still suffer from one or more chronic conditions which constrains their work performance [14]. Sickness absence remains a large problem in terms of costs, labour participation and social consequences [11,15,16]. The most common causes of sickness absence are: musculoskeletal complaints, mental health problems, and cardiovascular diseases [17,18]. Chronic diseases are more common among workers of 45 years and older, and it is expected that this age group will greatly increase, leading to a growing number of occupational health problems [17,18]. In the Netherlands, the total annual costs of absenteeism arising from health complaints and work disability amounted to 26 billion Euros, which accounts for almost 5% of the gross national product [14]. Additionally, Kremer and Steenbeek (2010) have computed that approximately 21.5% of absenteeism from work could be avoided if health services and employer and employee opportunities improved. To do this, shorter referral times, swifter medical care, improved collaboration between health care providers, reduced workload and an improved work-life balance are important factors [17,18].

### Return-to-work

Different return-to-work (RTW) interventions have been developed and evaluated to reduce sickness absence due to work disability. The effect, and occasionally the cost-effectiveness, of RTW interventions have been demonstrated in several observational studies [19-24], randomized controlled trials [25-30] and reviews [31,32]. For example, Franche et al. [31] concluded that work-based RTW interventions among employees with work-disability caused by musculoskeletal or other pain-related conditions can reduce work disability duration and associated costs. Lambeek et al. [33] showed that an integrated care programme for work-disabled participants with chronic low back pain has the potential to significantly reduce societal costs and improve care effectiveness, quality of life and function on a broad scale [33]. Other studies demonstrate that implementing preventive interventions before sickness absence occurs can be successful in preventing long-term sickness absence [19,29,34,35]. Whilst these results are very promising for society, participants, and employers, more research is necessary to confirm the long-term effects and cost-effectiveness of such interventions. However, methodological limitations in the current literature make it difficult to assess the feasibility of the different interventions proposed. These limitations include restriction of the population of interest due to the strict inclusion criteria, restrictions in the nature of the intervention evaluated, restrictions in study-design and the measurement of outcomes. There is moreover, a lack on transparency and consensus about how to conduct trial-based economic evaluations within the general field of employability.

### Objectives

This paper is the first of its kind to describe a transparent, uniform methodological design for the economic evaluation of several different trial-based interventions in the field of employability, disability and return-to-work. Known as the 'CASE-study' (Cost-effectiveness Analysis of Sustainable Employability), the project's aim is to evaluate at least five different employability interventions. All of the interventions share the common aim of reducing absenteeism and optimising the employability of employees with a known disability or those at risk of disability due to musculoskeletal complaints, mental health problems or a combination of both musculoskeletal and mental health complaints. In contrast with previous studies, the project design has no population restrictions and allows for the economic evaluation of any employability intervention regardless of the population they were developed for. Furthermore, the project also includes interventions provided in the primary and specialist health sectors as well as those delivered within the workplace. It also includes interventions aimed at reducing health care costs, wage replacement costs and other intervention costs in addition to improvements in return to disability duration and work return rates as measured by self-reported return to work dates, total duration of absence and subsequent recurrences, etc. Other outcomes include quality of life, quality of work life and general health status, etc. In addition, we cast a wide net to include a range of study designs since RCTs are not always possible in the economic evaluation of employability interventions. This paper describes the patient inflow in the event of an RCT or a quasi-experiment with a matched controlled design being conducted. For every employability intervention, an effect study will be conducted. In addition, from a societal perspective, a trial-based economic evaluation for every employability intervention will be carried out in a consistent manner. The results of these analyses will be discussed elsewhere in the future, but we hypothesize that the employability intervention is preferable in terms of costs, effects and utility compared to usual care. This paper will therefore demonstrate the strength of the CASE-study project in prescribing a methodological design which allows for the assessment of several different types of employability interventions within varying populations.

## Methodology

### Methods/design

#### Organisation of the CASE-study

**Figure 1:**
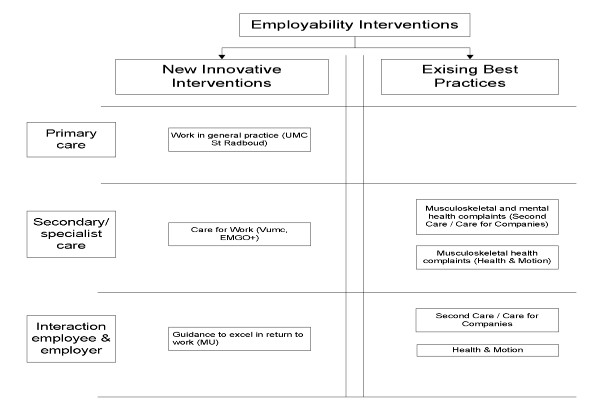
**Employability interventions included in the CASE-study**.

We will carry out an economic evaluation designed for at least five Dutch employability interventions. Those undertaking the evaluation are not connected with the organisations or individuals who have developed, or are in the process of developing the employability interventions so no competing interests arise. Within the CASE-study each existing or innovative intervention will be compared with usual care for work-disabled employees or those at risk of work disability due to medical restrictions. At the start of the CASE-study, nine programmes were interested in participating in the project. Unfortunately four programmes could not collaborate because they were unable to provide the minimum required number of participants eligible for the study, or could not provide a comparable control group. The remaining five employability interventions have the ability to comply to the minimal requirements and focus on primary care, secondary or specialist care, and/or on the interaction between the employer and employee. Two existing employability interventions (called 'Best Practices') and three newly developed employability interventions are included in the study. The employability intervention focussing on primary care aims to acknowledge the importance of employability in general practice. This intervention is under development at the University of Nijmegen (UMC St Radboud). The employability intervention in specialist care is being developed jointly by the VU University Medical Centre Amsterdam (VUMC) and the Institute for Health and Care Research (EMGO+) and focuses on employability among patients who suffer from rheumatoid arthritis. The third newly developed intervention aims at improving the interaction between the employee and his/her employer by appointing an independent mediator. The intervention is being developed by a separate team at Maastricht University. One of the 'Best Practice' interventions, developed by Health & Motion Nederland, offers patients with physical health complaints the ability to implement a range of exercises learned at the physiotherapeutic setting in their working environment by means of a workplace-intervention. The fifth and final intervention is developed by Second Care/Care for Companies (Best Practice) and aims at achieving sustainable employability among patients with physical and/or psychological health complaints using a multidisciplinary approach. An overview of these interventions is provided in Figure 1.

It is beyond the scope of this design article to describe the precise content of each employability intervention in detail. This will be done when the results of the economic evaluation per employability intervention are published. What is of importance in this paper is that the employability interventions use an innovative, systematic approach in order to support work participation in employees with health complaints. These are innovative because both the healthcare system and the private/work life of the employees are considered in the intervention. The features of the interventions differ according to the patient population, but the aim remains the same: to reduce sickness absence and increase the potential of achieving sustainable employability.

The protocol for this study has been approved by the Ethics Committee of Psychology (ECP107) at Maastricht University, the Netherlands.

#### Usual work resumption care in the Netherlands

The Gatekeeper Improvement Act stipulates that the responsibility for work resumption lies with the employer and the employee. The employer is obliged to start an employee's work resumption programme as quickly as possible, so the employee can resume his/her own work or other adequate work. In return, the employee should accept the work activities the employer provides. Furthermore, and unique in the world, the Dutch reintegration process provides the employer with salary and mandatory reintegration obligations for a period of two years [36]. Participants included in the care as usual group will continue to receive care for their health complaints, but attention to work-related factors influencing these will only occur minimally. Nevertheless, since attention to labour and health-related characteristics is obligatory under the Gatekeeper Improvement Act [36], participants in the care as usual group who are work-disabled will also receive adequate care (care as usual). Participants included in the control group will be treated and supervised according to good clinical practice based on the evidence-based guidelines of the Dutch association of occupational physicians [37,38].

#### Participants

Employees eligible for participation in the study will be work-disabled or at risk of work disability due to medical restrictions. In order to identify these employees, we will use employer registration forms, registrations with health care organizations and insurance companies, and registration forms from those providing the intervention. Employees eligible for inclusion will be 18 to 63 years of age, perform paid labour for at least 12 h per week, and are able to communicate in Dutch. Reasons for exclusion are absenteeism for 1.5 years or longer, fulltime students with a student job, informal caregivers and/or volunteers.

In order to recruit a sufficient number of eligible employees, one or more health care providers providing an employability intervention will screen service users for inclusion in the study. When appropriate, information on the research will be provided to the patient. This will be done orally by the health care provider, possibly supported by a handout containing contact information for the researchers, what is expected of participants, privacy concerns, and so on. A self-completion card will be attached to each flyer (handout) consisting of a short checklist for inclusion and informed consent form for signing. The employee is requested to fill in the short checklist, sign the informed consent form, and send these back to the researcher. Participants will have at least 1 week to consider their participation in the study before signing the informed consent form. Participants will be screened for exclusion by the researcher on the basis of answers provided on the checklist. If an employee meets all the selection criteria and is willing to sign the consent form, randomization or matching will then place. Before the intervention starts, the first (baseline) measurement occurs. An email with a login code is sent to all participants, and they will be asked to fill in the baseline questionnaire. The questionnaire for the intervention group is identical to the one for the control group.

#### Study design

**Figure 2:**
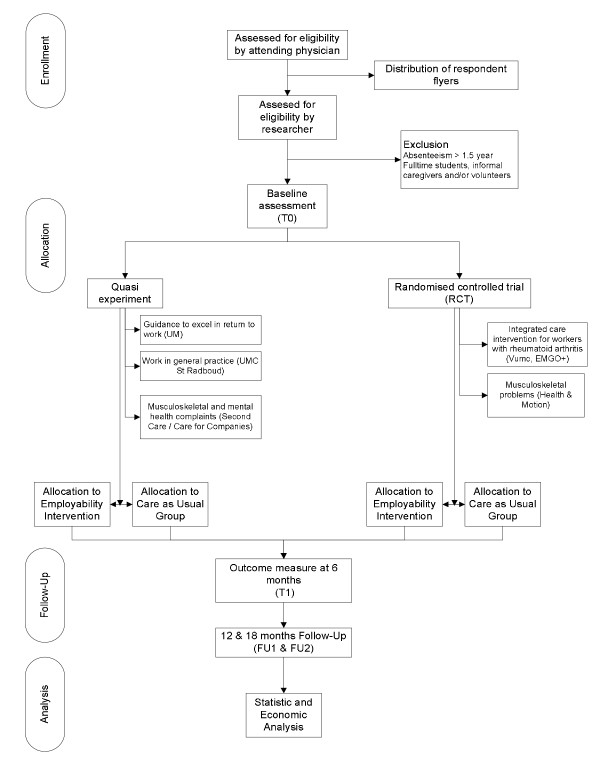
**Participants flow**.

As suggested by Drummond et al. (2001), a randomized controlled trial (RCT) remains the standard design for evaluating the relative effect of candidate interventions [39]. However, due to practical reasons, randomly assessing participants to the employability intervention or care as usual is not always possible in the CASE-study. Employers and occupational physicians are legally obliged to offer sickness absence guidance, and to do so, they refer employees to one or more employability interventions. Thus, a quasi experiment, comparing employees who are allocated to an experimental intervention in a non-random manner with those non-randomly assigned to a conventional intervention is regarded as the next best study design. Unlike an RCT, a quasi experiment consists of a control group derived from a large non-equivalent population. The variables are not manipulated, and participants are selected on the basis of matched criteria with those in the non-intervention group. Randomisation is not used therefore, and participants/control members are selected on the basis of their having an equal chance of demonstrating a causal relationship between the intervention and its outcomes for the control or experimental intervention. Both quasi experiments and RCTs are acceptable for the economic evaluation of employability interventions. The anticipated flow of subject enrolment in a quasi experiment or RCT is shown graphically in Figure 2.

#### Randomization/matching

In the case of randomization, the minimization method will be applied. Minimization aims to ensure an excellent balance for several prognostic factors in small groups [40]. Employees consenting to participate will be pre-stratified according to age (18-32 years, 33-48 years, 49-64 years), gender, and working hours (12-24 h/week, > 24 h/week). Since health care providers will treat participants in both the intervention and in the control groups, the study cannot be blinded. Matching i.e. formulating experimental and control groups on the basis of pairing participants on the basis of predetermined matching criteria will be used. These matched-criteria are age, working hours and health complaints. Thus, an employee in the intervention group will be matched to someone meeting the same criteria who will then act as a member of the control group.

#### Timeline of the study

During recruitment, employees will be asked whether they want to participate in the research by one or more health care providers operating in the employability intervention. Participation requires employees to fill in a self-reported online questionnaire at time points T0 (baseline), T1 (6 months after admission), FU1 (12 month follow-up) and FU2 (18 month follow-up). After 18 months the study results for sustainable employability and quality of life are expected to extend into subsequent years. The timeline of the study can also be found in Figure 2.

### Outcome assessment and data collection

The outcome assessment and data collection is identical for every employability intervention. Questions derived from different questionnaires, such as the 'National Working Conditions Survey' (NEA) [18], 'EuroQol 5 Dimensions 5 Levels' (EQ-5D-5 L) [41], 'Short Form Health Survey' (SF-36) [42], 'Trimbos/iMTA questionnaire for costs associated with psychiatric illness' (TIC-P) [43], and 'the productivity and disease questionnaire' (PRODISQ) [44], are combined into a retrospective self-reported questionnaire. The economic evaluation will be analysed from a societal perspective. This means that the most appropriate set of costs captured from the data (e.g. health care costs, productivity costs etc.), regardless of where the costs or benefits occur, will be applied [36].

### Effect evaluation

#### Primary outcome measure

The primary outcome measure in the CASE-study is reduced sickness absence duration in calendar days. Reduced sickness absence will be measured subjectively via patient self-report records. The duration of sick leave will be calculated as the time in calendar days since reduced productivity occurred (baseline). This will then be compared with the productivity time of a person at 6, 12, and 18 months. The success of an intervention will be defined by comparing the average reduction in sickness absence between the intervention group and the control group. The employability intervention is effective when the employees in the intervention group return to work twice as quickly as employees in the control group. Data on sickness absence duration will be gathered by using the productivity and disease questionnaire (PRODISQ) [45].

#### Secondary outcome measures

Data on secondary outcome measures will be gathered by means of self-administered questionnaires such as, NEA [18], EQ-5D-5 L [41], SF-36[42], TIC-P [43] and PRODISQ[44].

##### - Sustainable employability

After commencing a treatment intervention, a follow-up measurement will take place at 12 and 18 months to analyze the sustainability of employability. An intervention will be deemed to be unsuccessful when absenteeism of more than fourteen consecutive days after receiving the productivity intervention occurs. On the basis of previous research, it will be assumed that the long-term effect of the employability intervention stabilizes after 18 months [24].

##### - Self-rated health

Self-related health will be assessed using one item from the Short Form Health Survey (SF-36). This widely used measurement tool describes how a respondent values and assess his/her general health [46]. Data gathered via questions derived from the NEA [18] will be used to detect health complaints underlying work disability and co-morbidity.

##### - Quality of Life

An explorative analysis of productivity costs and quality of life showed a clear relationship between both variables [46]. Therefore, quality of life will be measured in work-disabled employees by using the EuroQoL five dimensions, five levels (EQ-5D-5 L) [41]. The EQ-5D-5 L comprises 5 dimensions: mobility, self-care, usual activities, pain/discomfort and anxiety/depression. There are five possible answers for each dimension, defining (5^5^) 3125 possible health states. Furthermore, the EQ-5D-5 L includes a visual analogue scale (VAS) rating from zero (worst imaginable health state) to 100 (best imaginable health state) [41].

### Economic evaluation

Self-reported questionnaires will capture the costs and benefits from a societal perspective, thus independently of those who bear the costs, and those who receive the benefits. Self-reported questionnaires appear to be a reliable alternative for cost-diaries, at least for a recall period of 6 months, since participants will still remember significant life events [45,47-49]. Costs that arise in different years will be discounted at 4% [50]. Costs directly related to the provision of the employability intervention will be objectively registered via the providers of the employability intervention. Direct healthcare costs and direct non-healthcare costs comprise respectively: costs of visits for primary and secondary care, home care, drugs, and for visits to an alternative or informal health therapist. Direct costs will be measured by an adapted version of the Trimbos/iMTA questionnaire for costs associated with psychiatric illness [43] and will be estimated on the basis of prices suggested in the cost calculation guidelines for healthcare in the Netherlands [50]. The prices of prescribed drugs will be based on Daily Defined Dosage (DDD) taken from the Royal Dutch Society for Pharmacy [50]. Indirect costs will be determined by measuring data on the costs of lost productivity due to work disability and are derived through the PRODISQ [46]. The indirect costs of production loss due to sick leave will be calculated by means of the friction cost method whereby the number of days of sickness absence and lost earnings are used to calculate the costs of production losses. Lost earnings will be determined on the basis of the differences between income at baseline and income at follow-up. Based on a mean added value of the Dutch working population, production losses are confined to the period needed to replace a sick employee [44]. The indirect costs of productivity losses without sick leave will be based on the number of days in which productivity was hindered due to health complaints and an estimation of the efficiency in these days [51]. Furthermore, compensation will be taken into account when determining production loss. Information about the number of hours to catch up with the work will be obtained as well as the way in which this compensation was achieved. If these data are considered invalid, for example because the income data of too many employees are unknown, we will use national data of average production value per worker, related to the education level and labour sector.

### Sample size

For the economic evaluation of the three newly developed employability interventions, the study sample size was pre-specified by the researchers and the developers of the particular interventions. For the sample size calculation of the existing 'Best Practices', we expect that a Hazard Ratio (HR) of 2.0 indicates a relevant difference in the average number of hours/days of lost work due to work disability between employees in the intervention group and employees in the control group after 18 months. This HR is based on findings of recent studies in occupational health care [25,52-55]. With a power of 80% at a 0.05 significance level, the sample size was calculated to be 120 employees [56]. Taking into account a dropout rate of 15%, 5% per follow-up measurement time, a total of 142 participants are needed. A dropout is defined as an employee who has not filled in the questionnaire at one or more measurement time-points.

### Data analyses

#### Effectiveness

All statistical analyses, performed to distinguish differences between the control group and the intervention group, will be performed according to the intention-to-treat principle. This means that the participants will remain in the group they were allocated to at baseline. All data regarding primary and secondary outcomes will be analysed using descriptive statistics. To control for protocol deviations likely to cause bias, data will be analyzed using per-protocol analyses whereby outcomes will be compared between those participants who received at least one or a partial intervention session and the control group. With the aim of correcting for possible regression to the mean, the baseline value of the particular outcome variable will be added to the model. Days of sick leave in the year before the inclusion, measured at baseline, will be added as a potential effect-modifier.

Diagnosis and prognostic parameters can be confounders, thus the Cox regression analysis for confounders will be used to adjust for differences between control and intervention groups. If necessary, differences in baseline will be adjusted for prognostic dissimilarities. Missing data will be handled using SPSS missing value analysis on item level. Completely missing measurements will be handled using multiple imputation.

The primary dependent variable is days of absence from work. In order to estimate the number of absence days, survival analyses will be used. There are two reasons why we opted to use survival statistics. Firstly, the time to sustainable employability is unlikely to be normally distributed which will make parametric statistical analysis difficult or inappropriate. Increasing productivity can occur quite early, possibly within months, but can also, as expected in the care as usual situation, may not be prolonged or may even decrease again. Secondly, survival analysis accounts for dropout by the technique of censoring. Participants who drop out will have the same hazard of sustainable employment than those that remained in the study. The Cox Proportional hazard model will therefore be used to analyse the HR of the days of sick leave duration. Reduced sickness absence duration in calendar days will be analysed with the Kaplan Meier survival method, and the differences in absence duration between both groups will be tested with a log rank test.

To analyse the secondary outcomes, longitudinal multilevel analyses will be used to assess the differences in health and quality of life between the control group and the intervention group.

Data will be analysed in SPSS 17.0.

### Economic evaluation

The economic evaluation will be carried out according to the intention-to-treat principle. Non-parametric bootstrapping (1000 replications) will be used to obtain 95% confidence intervals to estimate the uncertainty surrounding the cost differences, because cost-data are usually skewed to the right [57].

For the cost-effectiveness analysis, the incremental cost-effectiveness ratio (ICER) will be calculated by assessing the ratio of the differences in costs between the groups to the differences in effects between the groups. The ICER indicates whether the additional investments needed for the interventions gain at least one extra unit of effect compared with care as usual [57]. The cost-utility ratio will be calculated by the incremental costs divided by the difference in QALYs [39,58].

Bootstrapping with 5000 replications will be used to estimate the sample-uncertainty around the point estimates (ICER and ICUR). The bootstrapped ICERs and ICURs will be graphically presented on a cost-effectiveness plane. Furthermore, acceptability curves will show the probability that the employability intervention is cost-effective at a specific ceiling ratio, which is the maximum amount of money society is willing to pay to gain one extra unit of effect or a gain in quality of life [59]. Finally, the robustness of the different parameters on the cost-effectiveness calculation will be assessed by sensitivity analyses. Data processing will be done in SPSS 17.0 and bootstrapping will be carried out in Excel.

## Discussion

This study protocol provides detailed information on how to assess the effectiveness and cost-effectiveness of employability interventions among employees with work disabilities or employees at risk of becoming work-disabled due to health complaints. The results derived from these economic evaluations can benefit the individual employee, the employer and the broader occupational health practice sector because illness duration, care consumption and costs of lost productivity are expected to decrease if the employability intervention is cost-effective. For society as a whole, cost-effective employability interventions are attractive since they are expected to increase labour participation and public health. Furthermore, we feel that productivity is the key to higher economic growth as we are facing an ageing population.

A limitation of this study-design is the generalisability to other countries. The employability interventions are specifically tailored for the Dutch context in which they will be implemented. When applying these interventions in other countries, specific characteristics of the population in relation to the societal, political and cultural context in which the interventions are to be used should be taken into account. Although international generalisability might be restricted and require adaptation to the intervention, lessons can be learned and implementation advice given to those engaging in future work.

Selection bias cannot be ruled out in this design because we ask the healthcare provider to select employees to participate in our study. As a consequence, healthcare providers cannot be blinded. Furthermore, the consultation with the health care provider might change the participants' point of view on work disability. This 'Hawthorne effect' will be born in mind and might overestimate or underestimate the effect of the intervention. However, we feel that minor interactions such as the provision of information about the study will not be an important source of bias.

The strengths of this pragmatic design looking at the cost-effectiveness of employability interventions versus usual care, is the comparison under 'real life' circumstances of the intervention and control variables. Resource use is self-reported by participants and will be collected separately from the unit costs or prices of those resources. We will keep in mind the possibility that health care and intervention providers in different settings may wish to apply their own prices to the units of resource use. Furthermore, due to the standard recall period of 6 months, an adequate estimation of resource use is linear over time. This linear time trend restricts overestimation and underestimation of resource use between two consecutive measurement moments. An additional strength of this design is the inclusion of losses due to sick leave and the costs of using health care facilities together with productivity losses during working periods whilst unwell (presenteeism). In developing the methodology, we kept in mind the possibility that the employability and usual care interventions may have different effects on the component of reduced productivity while at work and therefore capture these. Besides internal validity, external validity of the results of the economic evaluation is reached by analysing the data from a societal perspective. We can split the calculated cost-effectiveness from the societal perspective to other perspectives, e.g. employee perspective, organisational perspective, etc.

To conclude, the research design provided in this article will be applied to each employability intervention included in the CASE-study. The CASE-study is the first study assessing the cost-effectiveness of different employability interventions. In addition, this design might contribute to new and better guidelines in the conduction of future economic evaluations in the occupational health sector. The results of the CASE-study will become available between 2012 and 2014.

## Abbreviations

CASE-study: Cost-effectiveness analysis sustainable employability; CEA: Cost-effectiveness analysis; CUA: Cost utility analysis; DDD: Daily defined doses; ECP: Ethics committee of psychology; EQ-5D-5 L: EuroQol 5dimensions 5levels; HR: Hazard ratio; ICER: Incremental cost effectiveness ratio; ICUR: Incremental cost utility ratio; NEA: National working conditions survey; PRODISQ: PROductivity and disease questionnaire; QALY: Quality of life; RCT: Randomized control trial; RTW: Return to work; SF-36: Short form (36) health survey; TIC-P: Trimbos/iMTa questionnaire for cost associated with psychiatric Illness; VAS: Visual analogue scale.

## Competing interests

The authors declare that they have no competing interests.

## Authors' contributions

CN is responsible for the data collection and drafted the manuscript. CN developed the study design. All authors participated in discussing the design of the study and developing the study protocol. All authors have read and corrected draft versions of the manuscript and approved the final manuscript.

## Pre-publication history

The pre-publication history for this paper can be accessed here:

http://www.biomedcentral.com/1471-2458/12/43/prepub
